# Derm-ographics: The Australian Dermatologist and Social Media

**DOI:** 10.2196/48975

**Published:** 2023-12-05

**Authors:** Antonia C Rowson, Saskia J Rowson

**Affiliations:** 1 The Alfred Hospital Melbourne Australia; 2 Austin Hospital Heidelberg Australia

**Keywords:** dermatology, social media, patient education, LinkedIn, Facebook, online presence, dermatologist, dermatologists, demographic, Twitter, X, YouTube, TikTok, ResearchGate, Instagram, provider, physician, technology use

## Abstract

Social media significantly affects how patients understand their health and choose their healthcare providers, yet Australian dermatologists have a limited online presence compared to their global peers.

## Introduction

Social media has become ubiquitous in modern life. Of 4.76 billion internet users worldwide, half use social media for 2.5 hours each day [1]. The ever-expanding use of these sites poses a relatively new consideration for doctors, especially in private practice. Evidence suggests patients have begun to rely on social media when choosing a clinician [2,3]. As a relatively visual specialty, dermatology lends itself well to social media. In this paper, we aimed to characterize the extent of online uptake by contemporary Australian dermatologists and to inform clinicians of their colleagues’ practices in regards to this emerging marketing and educational platform.

## Methods

We searched the Australian Health Practitioner Regulation Agency (AHPRA) register of practitioners for those listed on the Australian College of Dermatology (ACD) “Find a Dermatologist” service on August 1, 2022. Of the 411 ACD-listed dermatologists, 8 were no longer AHPRA registered, 6 were practicing outside of Australia, and 6 worked exclusively in the public health system; these 20 were excluded. Duration of practice, location of practice, and sex were taken from AHPRA data (Multimedia Appendix 1). Location of practice was then assigned as either metropolitan or rural in accordance with the Australian Government’s Modified Monash Model.

A Google search was done for each dermatologist identified, using their full professional name and the term “dermatologist.” Professional websites were used to identify practice size. Further searches of the following social media platforms were then performed: Facebook, X (formerly Twitter), Instagram, YouTube, ResearchGate, LinkedIn, and TikTok. Only publicly accessible, professional accounts were included in our analysis.

## Results

Professional social media use was not prevalent among the 391 Australia-based private dermatologists identified (Table 1). The most commonly subscribed platform for professional use was LinkedIn. Of the dermatologists analyzed, 212 (54.2%) did not have LinkedIn, 168 (43%) had an individual LinkedIn, 2 (0.5%) had a practice LinkedIn, and 9 (2.3%) had both an individual and practice LinkedIn. The next most commonly used platform was Facebook. In decreasing frequency of use followed ResearchGate, Instagram, X, YouTube, and TikTok, respectively. Only 1 (0.3%) Australian dermatologist had an individual TikTok.

**Table 1:** Proportion of Australian dermatologists with professional social media accounts (N=391).

	LinkedIn, n (%)	Facebook, n (%)	ResearchGate, n (%)	Instagram, n (%)	X, n (%)	YouTube, n (%)	TikTok, n (%)
No account	212 (54.2)	288 (73.7)	299 (76.5)	329 (84.1)	368 (94.1)	373 (95.4)	390 (99.7)
Individual	168 (43)	22 (5.6)	92 (23.5)	21 (5.4)	11 (2.8)	10 (2.6)	1 (0.3)
Practice	2 (0.5)	74 (18.9)	0 (0)	32 (8.2)	8 (2)	8 (2)	0 (0)
Both	9 (2.3)	7 (1.8)	0 (0)	9 (2.3)	4 (1)	0 (0)	0 (0)

**Figure 1:**
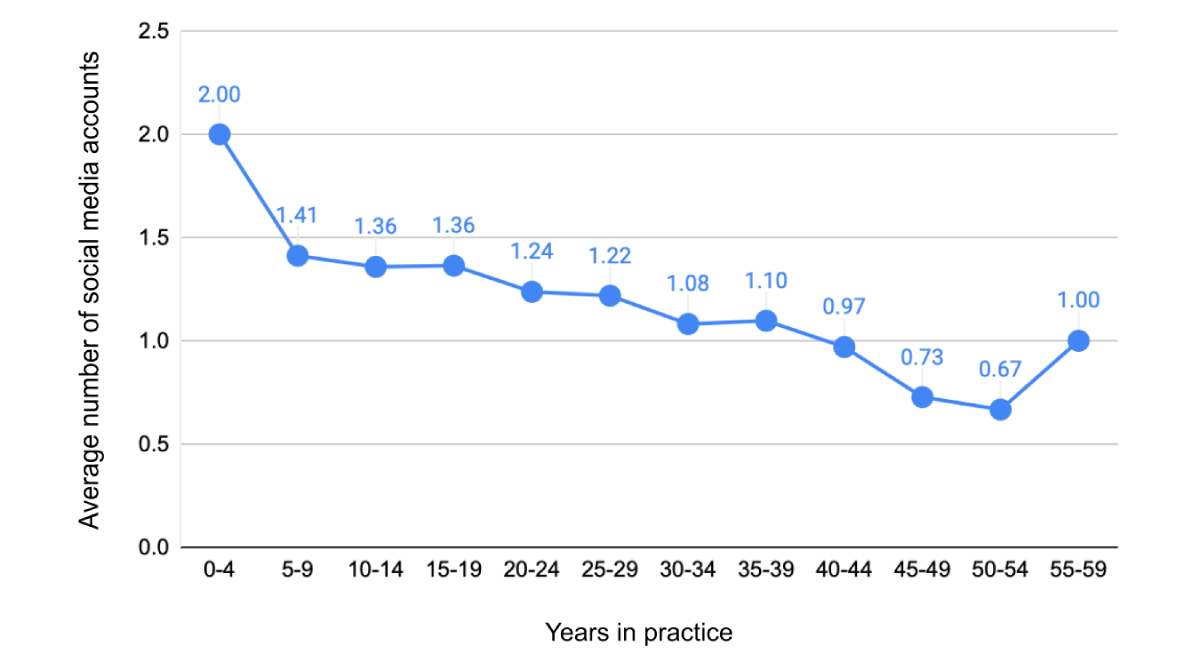
Average number of social media accounts by years in practice.

There were no significant differences in the average number of accounts by location of practice (*P*=.89) or sex (*P*=.34). While the mean number of social media accounts decreased with duration of practice, this trend did not reach statistical significance (*P*=.18) (Figure 1). Group practitioners, however, were more likely than sole practitioners to hold professional social media accounts (*P*=.003); group practitioners held on average 1.34 accounts versus 0.89 for those practicing alone.

## Discussion

Studies are mixed regarding the importance of social media to patients in selecting a dermatologist [2,3]; younger, less-educated patients, and those seeking cosmetic interventions are likely to rely more heavily on information available online [2]. Of surveyed American patients, 32% have used social media to make health care decisions [4]. The quality of such information remains a concern; as little as 5% of dermatologic content on Instagram is posted by qualified dermatologists [5,6]. Similarly, only 27% of keratosis pilaris content on TikTok is created by dermatologists [7]. There is evidence that most Saudi and American dermatologists plan to increase their social media presence [3,8]. We found that Australian dermatologists, regardless of most demographic influences, have a limited online presence, with an average 1.21 accounts per individual, and with no social media platform attracting more than half the overall group.

American dermatologists rate Instagram as their most “valuable” platform, followed by Facebook [8]. We conversely found that LinkedIn was the most subscribed platform among our cohort, followed by Facebook. Given the nature of these sites, this implies a greater Australian uptake of social media for professional networking among medical colleagues, rather than for patient-oriented promotional or educational endeavors.

In summary, social media is an underused avenue among Australian dermatologists, with international data suggesting the public is increasingly informed in their medical decision-making by online content, including practitioner selection. A majority of Australian dermatologists do not use social media for professional purposes, although the most prevalent platform, LinkedIn, is used by 1 in 2 dermatologists. LinkedIn and ResearchGate are typically used by individuals, presumably for academic promotional purposes, and Facebook and Instagram by practices, presumably for client engagement. This fact may inform readers’ uptake according to their intentions around the type of publicity.

## Appendix

Multimedia Appendix 1 Demographic details of included Australian dermatologists.

## Abbreviations

ACD: Australian College of Dermatology
AHPRA: Australian Health Practitioner Regulation Agency
