# Supplementary material for: Derm-ographics: The Australian Dermatologist and Social Media
Source: JMIR Dermatol. 2023 Dec 5;6:e48975. doi: 10.2196/48975 (PMC10731548; doi:10.2196/48975)
Supplement: Multimedia Appendix 1 [file derma_v6i1e48975_app1.docx]

*Appendix*

**Table S1.** Demographic Details of Included Australian Dermatologists.*

| *Parameter* | *Number* |  |
| --- | --- | --- |
| No. of dermatologists | 397 |  |
| Gender |  |  |
| Male | 203 (51.1) |  |
| Female | 194 (48.9) |  |
| Geographic location |  |  |
| Metropolitan | 351 (88.4) |  |
| Regional | 22 (5.5) |  |
| Both | 24 (6.0) |  |
| State/territory |  |  |
| NSW | 143 (36.0) |  |
| Victoria | 100 (25.2) |  |
| Queensland | 70 (17.6) |  |
| WA | 37 (9.3) |  |
| SA | 34 (8.6) |  |
| Tasmania | 5 (1.3) |  |
| ACT | 4 (1.0) |  |
| NT | 1 (0.3) |  |
| Multiple | 3 (0.8) |  |
| Duration of practice |  |  |
| 0-4 years | 1 (0.3) |  |
| 5-9 years | 17 (4.3) |  |
| 10-14 years | 95 (23.9) |  |
| 15-19 years | 66 (16.6) |  |
| 20-24 years | 38 (9.6) |  |
| 25-29 years | 55 (13.9) |  |
| 30-34 years | 37 (9.3) |  |
| 35-39 years | 31 (7.8) |  |
| 40-44 years | 33 (8.3) |  |
| 45-49 years | 11 (2.8) |  |
| 50-54 years | 12 (3.0) |  |
| 55-59 years | 1 (0.3) |  |
| Practice arrangement |  |  |
| Sole practitioner | 87 (21.9) |  |
| Group < 5 | 130 (32.7) |  |
| Group ≥ 5 | 169 (42.6) |  |
| Public only | 5 (1.3) |  |
| Retired/inactive | 5 (1.3) |  |
| Research only | 1 (0.3) |  |

*Values are given as number (percentage) unless otherwise indicated.
